# The expression, localisation and interactome of pigeon CRY2

**DOI:** 10.1038/s41598-021-99207-x

**Published:** 2021-10-13

**Authors:** Spencer D. Balay, Tobias Hochstoeger, Alexandra Vilceanu, E. Pascal Malkemper, William Snider, Gerhard Dürnberger, Karl Mechtler, Stefan Schuechner, Egon Ogris, Gregory C. Nordmann, Lyubov Ushakova, Simon Nimpf, David A. Keays

**Affiliations:** 1 Research Institute of Molecular Pathology (IMP), Vienna Biocenter (VBC), Campus Vienna Biocenter 1, 1030 Vienna, Austria; 2 Vienna BioCenter PhD Program, Doctoral School of the University of Vienna and Medical University of Vienna, 1030 Vienna, Austria; 3 Max Planck Research Group Neurobiology of Magnetoreception, Center of Advanced European Studies and Research (Caesar), Ludwig-Erhard-Allee 2, 53175 Bonn, Germany; 4 Department of Neuroscience, Johns Hopkins University School of Medicine, Baltimore, USA; 5 Institute of Molecular Biotechnology of the Austrian Academy of Sciences (IMBA), Vienna Biocenter (VBC), Dr. Bohr-Gasse 3, 1030 Vienna, Austria; 6 Monoclonal Antibody Facility, Max Perutz Labs, Medical University of Vienna, Dr. Bohr-Gasse 9, 1030 Vienna, Austria; 7 Department of Anatomy and Neuroscience, University of Melbourne, Parkville, Australia; 8 Division of Neurobiology, Department Biology II, Ludwig-Maximilians-University Munich, Planegg-Martinsried, 82152 Munich, Germany

**Keywords:** Cellular neuroscience, Molecular neuroscience, Visual system

## Abstract

Cryptochromes (CRY) are highly conserved signalling molecules that regulate circadian rhythms and are candidate radical pair based magnetoreceptors. Birds have at least four cryptochromes (CRY1a, CRY1b, CRY2, and CRY4), but few studies have interrogated their function. Here we investigate the expression, localisation and interactome of clCRY2 in the pigeon retina. We report that clCRY2 has two distinct transcript variants, *clCRY2a*, and a previously unreported splice isoform, *clCRY2b* which is larger in size. We show that *clCRY2a* mRNA is expressed in all retinal layers and *clCRY2b* is enriched in the inner and outer nuclear layer. To define the localisation and interaction network of clCRY2 we generated and validated a monoclonal antibody that detects both clCRY2 isoforms. Immunohistochemical studies revealed that clCRY2a/b is present in all retinal layers and is enriched in the outer limiting membrane and outer plexiform layer. Proteomic analysis showed clCRY2a/b interacts with typical circadian molecules (PER2, CLOCK, ARTNL), cell junction proteins (CTNNA1, CTNNA2) and components associated with the microtubule motor dynein (DYNC1LI2, DCTN1, DCTN2, DCTN3) within the retina. Collectively these data show that clCRY2 is a component of the avian circadian clock and unexpectedly associates with the microtubule cytoskeleton.

## Introduction

Cryptochromes are blue-light absorbing flavoproteins that are related to DNA photolyases and conserved across many taxa^1^. In plants, photosensitive cryptochromes regulate developmental processes^2^ and in *Drosophila*, cryptochromes act as photoreceptors that entrain the circadian clock^3^. Photosensitive animal cryptochromes (Type 1) absorb light through binding of the cofactor flavin adenine dinucleotide (FAD) chromophore^4,5^, unlike vertebrate cryptochromes such as mammalian CRY1 and CRY2 (Type 2)^6^. These Type 2 cryptochromes primarily function as light-independent transcriptional regulators of the circadian clock^7–10^. More recently, Type 4 cryptochromes have been characterized in fish, frogs, reptiles, and birds^11^. These molecules do not appear to play a role in circadian transcriptional regulation^12–14^ and have retained photosensitivity through FAD binding^15–18^. Photoabsorption induces electron transfer between FAD and a chain of highly conserved tryptophan residues, which creates radical pairs that are predicted to be sensitive to the external magnetic field^19,20^. For this reason, Type 4 cryptochromes are considered to be the best candidate molecules to mediate a radical pair based mechanism of magnetoreception in birds^16,17,21,22^.

Avian species have at least four cryptochrome isoforms: CRY1a, CRY1b, CRY2 (Type 2) and CRY4 (Type 4). Each exhibit a high degree of homology with mammalian CRY1 and CRY2, except for the variable C-terminus^6,17^. A number of previous studies have focused on CRY1a/b and CRY4, but the role of CRY2 has largely been ignored^21–24^. We have recently shown that pigeon (*Columba livia*) clCRY2a does not bind FAD^21^, suggesting it is not light-sensitive and is unlikely to participate in the primary generation of radical pairs sensitive to magnetic fields. Additionally, the expression of *CRY2* in the pigeon, European robin, chicken and zebra finch show circadian oscillations in the retina^12,21,22,25,26^, which indicates it may be involved with or regulated by the core circadian clock system. Pigeon *clCRY2a/b* mRNA levels peak at midnight ZT = 17 (00:00 CET), and decrease during the day to its lowest levels in the afternoon at ZT = 8 (13:00 CET)^21^. In several species including the pigeon, the cyclic expression of *CRY2* is opposed by day-time dominant *CRY1* mRNA^11,21,22,26^, suggesting temporally specific roles for CRY1 and CRY2. In this manuscript, we describe the expression, localisation and interaction partners of pigeon clCRY2a/b in efforts to understand its function in the retina. These data show that clCRY2a/b interacts with core components of the circadian clock and multiple proteins associated with the microtubule cytoskeleton.

## Results

### Cloning and retinal expression of *clCRY2a/b*

**Figure 1:**
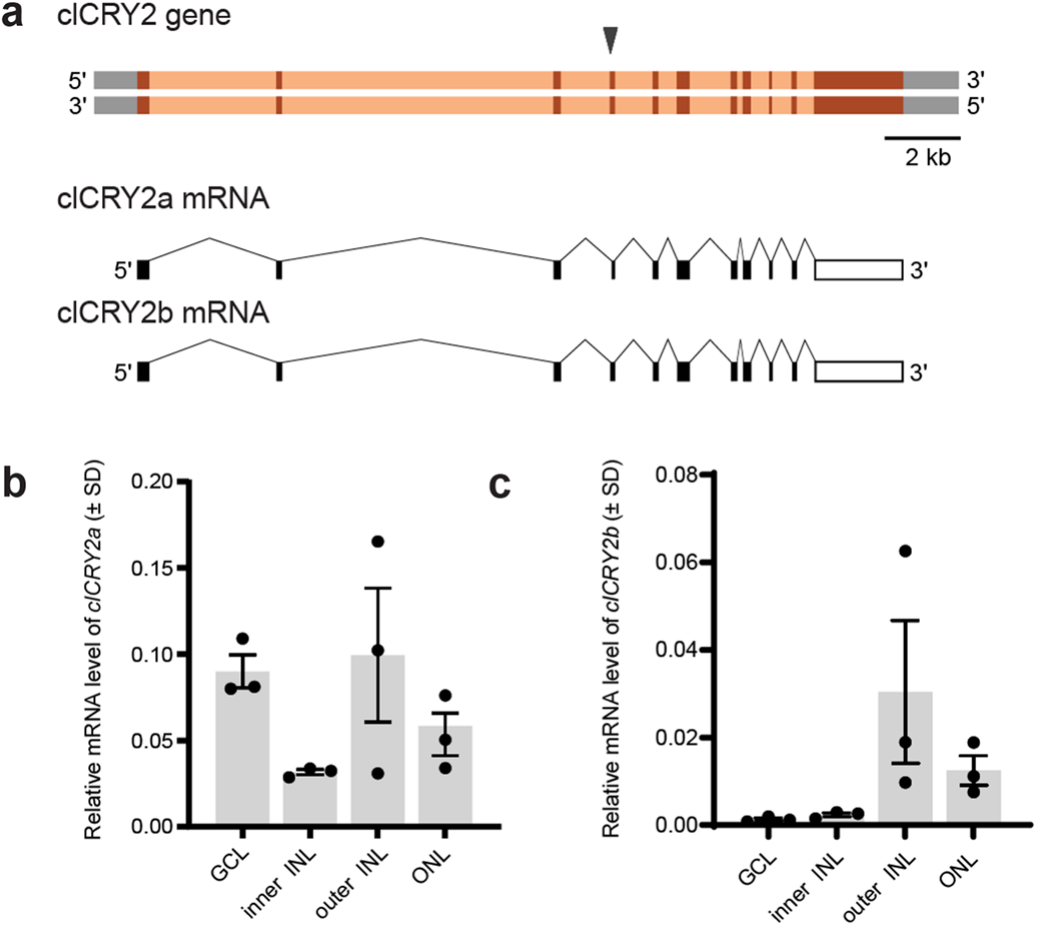
Cloning and retinal expression of clCRY2. (**a**) Genomic structure of the *Columba livia CRY2* gene and its transcripts, *clCRY2a* (10 exons, 1752 nt, 583 aa) and *clCRY2b* (10 exons, 1788 nt, 595 aa). The alternative splice site in exon 4 is indicated with a black arrowhead. Exon: red box, Intron: orange box, CDS: black box, UTR: white box. (**b**,**c**) Quantitative analysis of *clCRY2a* and *2b* expression by qPCR in micro dissected retinal layers collected at midday (n = 3 birds). *CRY2a* is expressed in all retinal layers, while *CRY2b* is enriched in the outer INL and ONL. *CRY2a* is expressed at higher levels relative to *CRY2b*. mRNA expression is normalized against the geometric mean of three control genes (*clHPRT*, *clGAPDH*, and *clTFRC*). Error bars: Standard Deviation (SD).

To clone pigeon *CRY2* we generated adaptor ligated cDNA libraries from adult pigeon retinae (n = 3) and performed rapid amplification of cDNA ends (RACE) using transcript specific primers designed from published avian *CRY2* sequences^27–29^. We identified a previously unreported splice isoform of *Columba livia CRY2*, which we named *clCRY2b*. Compared to *clCRY2a, clCRY2b* is characterized by an alternative splice acceptor site, which alters the 5′ boundary of the downstream exon 4, resulting in the insertion of 36 nucleotides (nt) (Fig. 1a, *clCRY2a* exon 4 = 89 nt, *clCRY2b* exon 4 = 125 nt). Both *clCRY2a* and *clCRY2b* mRNA sequences have been deposited in GenBank under the accession numbers KX168609.1 and KX168610.1, respectively. To determine if the two transcript variants had similar spatial expression profiles in the retina, we performed qPCR on laser dissected retinal layers (n = 3 birds) (Fig. S1a). We found that *clCRY2a* mRNA is present in all retinal layers, including the ganglion cell layer (GCL), inner portion of the inner nuclear layer (iINL), outer portion of the inner nuclear layer (oINL) and the outer nuclear layer (ONL) (Fig. 1b). *clCRY2b* is expressed approximately 15-fold lower than *clCRY2a* mRNA (Fig. S1b) and is present at low levels in the oINL and ONL (Fig. 1c).

### clCRY2a/b antibody design and validation

**Figure 2:**
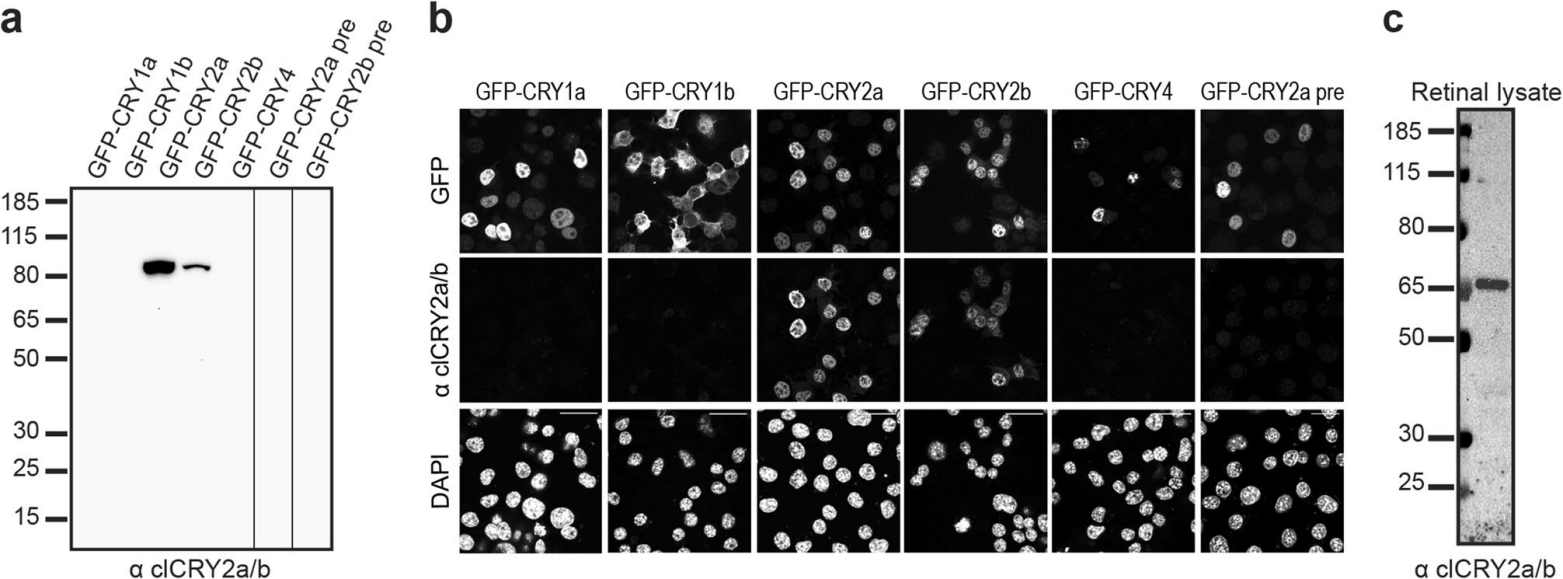
Generation and validation of clCRY2a/b monoclonal antibody. An antibody against the C-terminal (C516-V583) of clCRY2a/b was generated in mice. (**a**) Western blot of pigeon embryonic fibroblast (PEF) cell lysates overexpressing GFP-tagged clCRY1a, clCRY1b, clCRY2a, clCRY2b and clCRY4 incubated with α clCRY2a/b 2A3 reveals specific recognition of both clCRY2a and clCRY2b. No cross reactivity between the other cryptochromes was observed. Preincubation of the antigen with the antibody abolished any visible signal. (**b**) Immunostaining of α clCRY2a/b on GFP-tagged recombinant clCRYs expressed in N2A cells shows specific clCRY2a/b signal, as observed in western blots (**a**). (**c**) Western blot of purified α clCRY2a/b on pigeon retinal lysates confirms specificity, with recognition of a single ~ 67kDA band. Scale bar: 20 µm. See Fig. S2 for uncropped blots.

To study the localisation of clCRY2a/b in more detail, we designed, generated and carefully validated a monoclonal antibody against clCRY2a/b using an antigen in the C-terminal region that is present in both clCRY2a and clCRY2b (C516-V583), but variable in other cryptochrome isoforms. This peptide was expressed in *E. coli*, purified, and injected into BALB/c mice. The spleens of immunized mice were harvested, hybridomas generated, and individual clones screened by western blot analysis as previously described^21^. This resulted in the identification of clone 2A3, which was used to generate purified antibodies. To assess the specificity of the 2A3 antibody, we recombinantly expressed clCRY1a, clCRY1b, clCRY2a, clCRY2b and clCRY4 tagged to GFP in a pigeon embryonic fibroblast cell line (Figs. 2a,b, S2a,b). Western blot and immunofluorescence experiments confirmed that α clCRY2a/b 2A3 binds to clCRY2a and clCRY2b, and does not cross react with clCRY1a, clCRY1b or clCRY4 (Fig. 2a,b). In addition, pre-incubation of clCRY2a/b 2A3 with the antigen abolished immunoreactivity in both western blots and cell culture (Fig. 2a,b). To determine whether the clCRY2a/b 2A3 antibody recognises clCRY2a/b at endogenous levels, we harvested pigeon retinae at midday, prepared lysates and undertook western blot analysis (Figs. 2c, S2c). This resulted in a single band at approximately 67 kDa, which is consistent with the size of clCRY2a, the dominant isoform of clCRY2a/b.

### clCRY2a/b is broadly expressed in the pigeon retina

**Figure 3:**
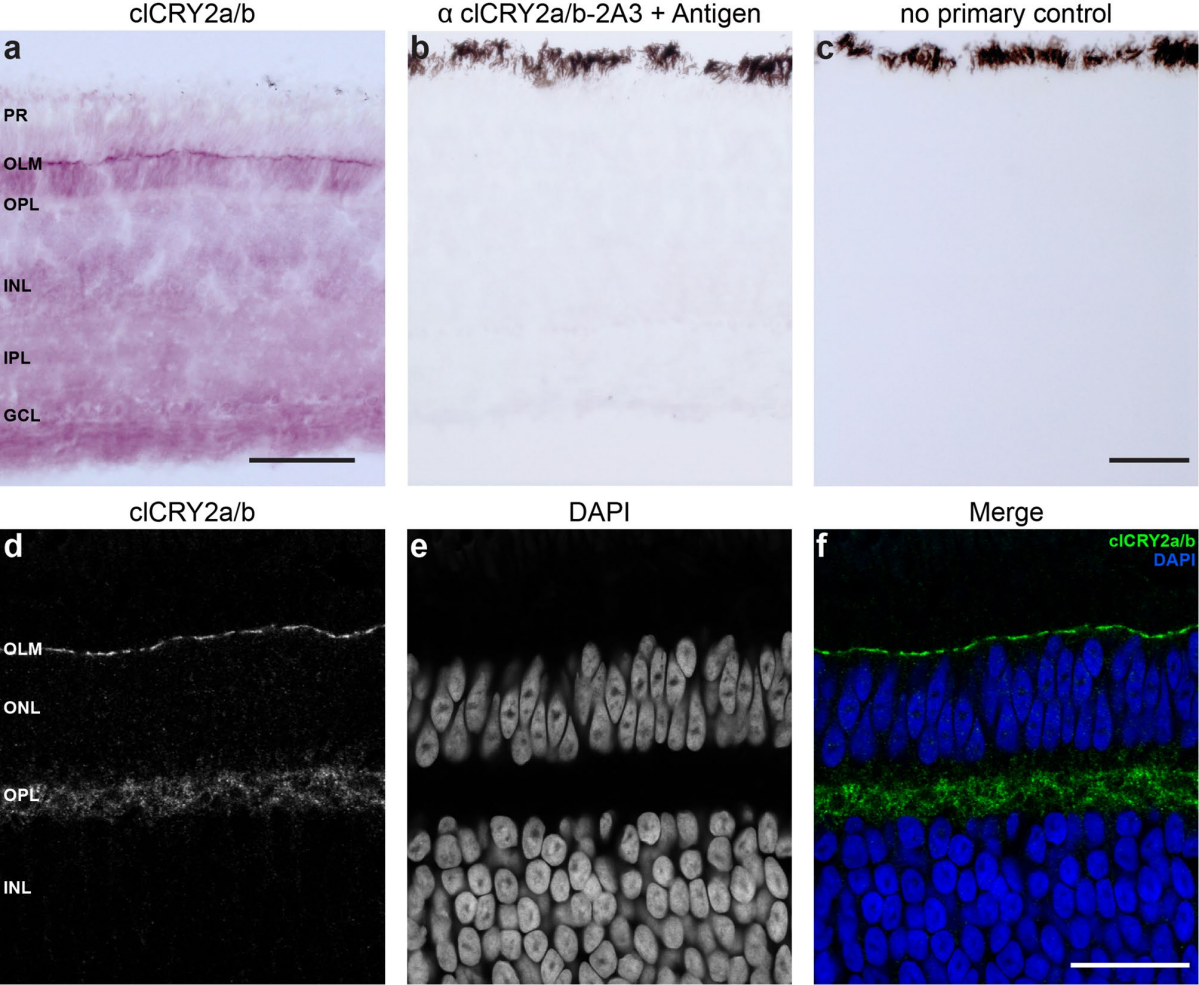
Localisation of clCRY2a/b in the pigeon retina. (**a**–**f**) Immunohistochemistry using the monoclonal clCRY2a/b antibody on adult pigeon retina harvested at midday (n = 3 birds). (**a**) Permanent staining reveals clCRY2a/b is localised throughout the retina and is enriched in the outer limiting membrane (OLM). (b) No staining is seen when 2A3 α clCRY2a/b is pre-incubated with the antigen. (**c**) Pigeon retinal sections treated with no primary clCRY2a/b antibody do not show any signal. (**d**–**f**) Immunofluorescence shows clCRY2a/b is enriched in the OLM and the outer plexiform layer (OPL). PR: photoreceptors, OLM: outer limiting membrane, ONL: outer nuclear layer, OPL: outer plexiform layer, INL: inner nuclear layer, IPL: inner plexiform layer, GCL: ganglion cell layer. Scale bars show 50 μm.

**Figure 4:**
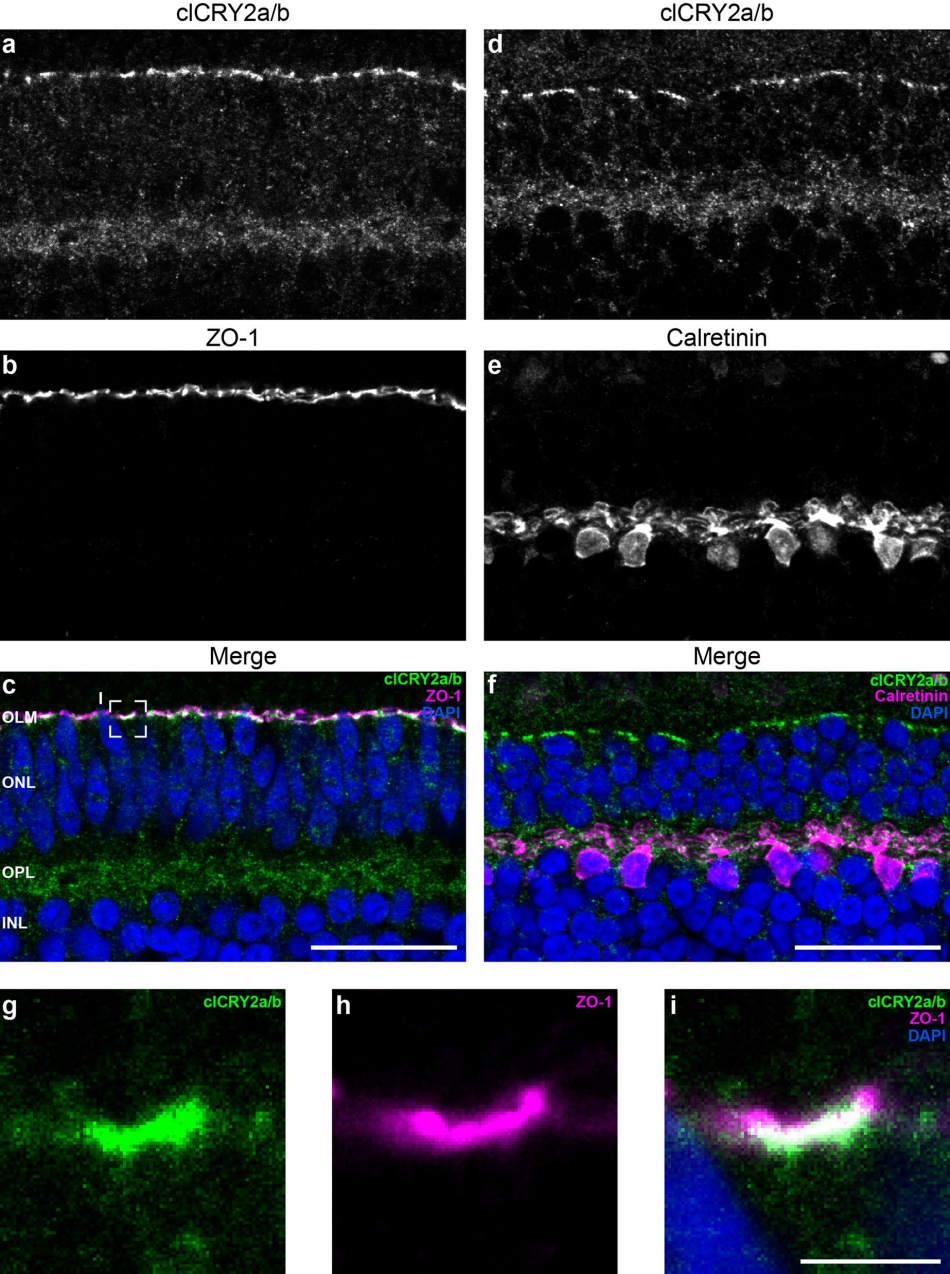
clCRY2a/b is enriched in the OLM and OPL at midday. (**a**–**i**) Double immunofluorescence staining reveals clCRY2a/b is expressed in the OLM (**a**–**c**) and OPL (**d**–**f**) at 12:00 CET. (**e**,**f**) Staining with Calretinin shows that clCRY2a/b is present within the cytosol of horizontal cells. (**g**–**i**) In the OLM, clCRY2a/b staining strongly overlaps with the scaffolding protein zonula occludens-1 (ZO-1), an adherens junction marker. OLM: outer limiting membrane, ONL: outer nuclear layer, OPL: outer plexiform layer, INL: inner nuclear layer. Scale bars: (**a**–**f**) 25 μm, (**g**–**i**) 3 μm. See Fig. S3 for staining controls.

Next, we investigated CRY2s spatial localisation in the avian retina employing the validated 2A3 antibody. Permanent staining of the pigeon retina (n = 3, collected at midday) showed that clCRY2a/b is present in all retinal layers with a notable enrichment in the outer limiting membrane (OLM) (Fig. 3a). This staining was abolished when preincubating the 2A3 antibody with the antigen, and in the absence of the primary antibody (Fig. 3b,c). High-resolution confocal imaging confirmed the distinct staining of clCRY2a/b in the OLM (Fig. 3d–f) which is reminiscent of adherens junctions proteins that link the inner segment of photoreceptors to the apical processes of Müller glia cells^30^. To further explore this, we performed immunohistochemistry with an antibody that binds to Zonula occludens-1 (ZO-1), a well-characterized intercellular component of adherens and tight junctions (Fig. 4a–c). In the OLM, ZO-1 and clCRY2a/b co-localised, with both proteins patterned as distinct stripes above the outer nuclear layer (Fig. 4g–i). This signal was not present in negative controls where sections were incubated without the primary antibody (Fig. S3a–c). These results suggest that clCRY2a/b co-localises with ZO-1 in the OLM, potentially part of a junction protein complex. Additionally, we double-stained clCRY2a/b with Calretinin, which labels brush-shaped H1 and H3 horizontal cells in the OPL^31^. This showed that clCRY2a/b is localised within the cytoplasm of horizontal cells (Fig. 4d–f). To assess if the localisation of clCRY2a/b changes over time we performed immunohistological staining on pigeon retina collected at midnight (n = 3, Fig. S4a–i). These findings mirror the results at midday, with clCRY2a/b staining present in the OLM overlapping with ZO-1 (Fig. S4a–c and g–i), and cytosolically in horizontal cells labelled with Calretinin (Fig. S4d–f). At both time points we observed faint punctate staining within the nuclei of cells within the ONL. We conclude that pigeon clCRY2a/b is broadly expressed in the retina and is enriched in the OLM and OPL during the day and night.

### The clCRY2a/b interactome

**Figure 5:**
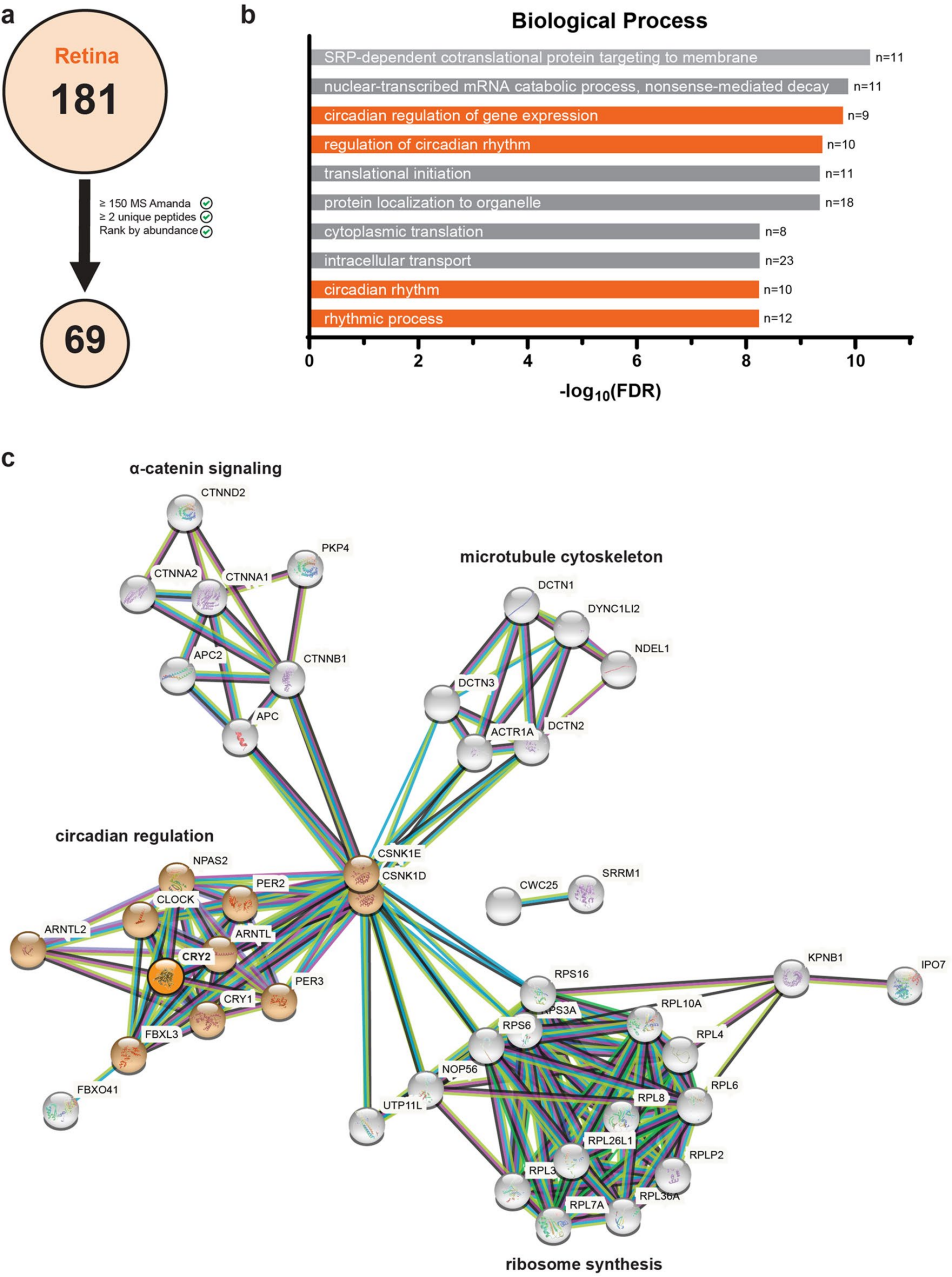
Putative clCRY2a/b interaction partners at midday. (**a**) Schematic depicting putative clCRY2a/b interactors coimmunoprecipitated with the 2A3 clCRY2a/b antibody from pigeon retina harvested at midday (12:00 CET). In total, 181 putative clCRY2a/b interactors that were not present in samples incubated with a control GFP antibody were pulled down from retinal lysates. Hits were ranked by average abundance and filtered for having an Amanda MS score of > 150 and 2 or more unique peptides. After this filtering strategy was preformed, 69 hits were identified in the retina. (**b**) Gene ontology (GO) analysis of clCRY2a/b and 68 putative clCRY2a/b interactors revealed an enrichment of proteins associated with multiple circadian rhythm GO-terms (orange). The top ten GO-terms based on FDR significance are shown. (**c**) STRING analysis (Version 11.0b, https://string-db.org/, STRING Consortium 2020^59^) shows clCRY2a/b interactors cluster in four distinct groups. Hits containing the circadian GO-terms highlighted in (**b**) are colored orange.

To further understand clCRY2s role in the retina and identify potential interacting proteins, we performed coimmunoprecipitation experiments coupled with mass spectrometry (CoIP-MS) on pigeon retinal lysates with our clCRY2a/b 2A3 antibody and a control GFP antibody (n = 3 retinas, collected at midday). Utilizing a previously established CoIP-MS pipeline^21^, we pulled down clCRY2a/b and 68 putative clCRY2a/b interacting proteins from retinal lysates incubated with our clCRY2a/b antibody (69 proteins total). Importantly, these hits were not found in the retinal lysate sample incubated with the control GFP antibody (Fig. 5a). Additionally, clCRY2a/b ranked sixth based on protein abundance after filtering for hits with 2 or more peptides (see Supplementary Table 1), confirming the validity of this approach.

Gene Ontology (GO) analyses revealed 132 significantly enriched GO-terms from potential clCRY2a/b interactors (See Supplementary Table S2), which included several circadian related terms such as “circadian regulation of gene expression”, “regulation of circadian rhythm”, “circadian rhythm” and “rhythmic process” (Fig. 5b). Application of the functional protein association network tool STRING revealed that clCRY2a/b interactors group into four main clusters: (1) α-catenin signaling; (2) components of the microtubule cytoskeleton; (3) circadian regulation; and (4) ribosome synthesis (Fig. 5c). In the circadian rhythm cluster, major constituents of the avian circadian clock were found to interact with clCRY2a/b, including CRY1, PER2, PER3, NPAS2, CLOCK and ARNTL (also referred to as BMAL1). Additionally, FBXL3 and FBXL21, the E3 ubiquitin ligases that target cryptochromes for cyclic degradation^32,33^, coimmunoprecipitated with our clCRY2a/b antibody. These findings suggest that clCRY2a/b is likely involved in the clock transcriptional feedback loop^34^. In addition, clCRY2a/b appears to interact with multiple components of the microtubule cytoskeleton, specifically those that are involved in dynein-mediated transport on microtubules. These hits include the minus-end microtubule motor dynein (DYNC1LI2), dynactins that tether dynein to microtubules (DCTN1, DCTN2 and DCTN3) and NDEL1, which positively regulates dynein (Fig. 5c). The third cluster identified by STRING analysis contained the microtubule stabilizing proteins APC and APC2, along with multiple α-catenins (Fig. 5c) which are associated with adherens junctions^35^. To explore if clCRY2a/b’s interactome changed over time we preformed CoIP-MS on retinas collected at midnight (n = 3, Fig. S5). Application of the same filtering strategy identified 87 putative interactors, with clCRY2a/b ranked fifth based on abundance (Fig. S5A, Supplementary Tables S3, S4). Many of these interactors were also found in the midday dataset, including core circadian proteins (CRY1, PER2, CLOCK, ARNTL), dynactins and dynein (DCTN1, DCTN2, DCTN3, DYNC1LI2) and α-catenins (CTNNA1, CTNNA2). Additionally, members from the AP-2 and AP-3 adaptor complexes (AP3D1, AP2A2) formed a fifth cluster in the midnight dataset. Overall, these data suggest that clCRY2a/b likely has multiple roles in the retina, through its interaction with stereotypical circadian clock proteins and molecules associated with the microtubule cytoskeleton.

## Discussion

In this manuscript we have studied the expression, localisation and interactome of pigeon CRY2, with a focus on the retina. We report the presence of two isoforms of clCRY2 (clCRY2a, and clCRY2b) that differ in size by 12 amino acids. qPCR analysis revealed that *clCRY2a* mRNA is expressed in all retinal layers whereas *clCRY2b* is present at lower levels predominantly in the inner and outer nuclear layer. These results mirror those of Bailey and colleagues who employed radioactive in situ hybridisation in the chicken, and reported broad *ggCRY2* expression in all retinal layers^27^. More recently, a cell atlas of the developing chicken retina was generated using single-cell RNA-Seq^36^. Consistent with our data, *CRY2* was detected in all subtypes of cones, rods, horizontal cells, bipolar cells, and retinal ganglion cells.

To further characterize clCRY2, we generated and validated a monoclonal antibody that detects both clCRY2 isoforms. Exploiting this resource, we report the presence of the clCRY2a/b protein in all major layers in the retina, with an enrichment in the OLM and the OPL at both midday and midnight. We recently defined the localisation of clCRY4 in the pigeon retina employing histological methods, and similar to clCRY2a/b, observe enrichment in the OLM and OPL^21^. In the OPL clCRY2a/b displays broad staining within the cytoplasm of horizontal cells, whereas clCRY4 is specifically localised to synapses between horizontal cells and photoreceptors. In the OLM, clCRY2a/b co-localises with the junction protein ZO-1 in a stereotypical pattern above the nuclei of the photoreceptors^35,37,38^. A similar pattern was observed for clCRY4, but in the case of clCRY2a/b the staining was more pronounced. Interestingly, Bolte and colleagues have reported strong OLM staining of CRY1a in the retina of the European robin using fluorescent immunohistochemistry^39^. These studies combined with our clCRY2a/b findings suggest that the avian OLM could host several CRYs. It would be interesting to determine if CRY2 is localised in the OLM of other bird species, and if this feature is also present in other non-mammalian vertebrates.

To explore the molecular function of clCRY2 in the retina, we performed coimmunoprecipitation coupled with mass spectrometry on retinal lysates collected at midday and midnight. For both timepoints we were able to effectively pull down clCRY2 and known CRY2 interactors such as PER2, CLOCK and ARNTL (BMAL1). Many of the hits were major components of the vertebrate circadian clock, including the E3 ligases FBXL3 and FBXL21, which regulate the circadian turnover of CRY2^32,33^. Based on its cyclic expression pattern^21^ and circadian-enriched interactome, our data supports clCRY2a/b as a component of the avian circadian clock transcriptional system^34,40^. Given that both positive (CLOCK and ARNTL) and negative (CRY1 and PER2) regulators of the circadian clock feedback loop were pulled down with clCRY2a/b, our data implies that clCRY2a/b participates as part of the repressive clock complex that binds and regulates the transcriptional output of the activation complex^33^. The regulation of the retinal clock is particularly important in birds such as pigeons, as the hormone melatonin is produced locally in photoreceptors^41–43^. Melatonin influences several visual processes including outer segment disc shedding^44,45^ and firing amplitude of the electroretinogram (ERG)^46,47^. The synthesis of melatonin is mediated by transcription of the E-box containing clock gene arylalkylamine N-acetyltransferase (AANAT), which is regulated by CRY/PER and CLOCK/BMAL1 activity^48,49^. Whether CRY2 plays a role in this process in the pigeon is unknown, but our data suggests clCRY2a/b at least has the molecular propensity to participate in the regulation of CLOCK/BMAL1 output.

Our CoIP-MS experiments suggest that clCRY2a/b is functionally promiscuous as proteins involved in translation initiation (RPL4, RPL6, RPL8), catenin signaling (CTNNA1, CTNNA2) and organization of the microtubule cytoskeleton^50^ (DCTN1, DCTN2, DCTN3, DYNC1LI2) were coimmunoprecipitated in retinal lysates collected both at midday and midnight. The latter are of interest as DYNC1Ll2 has been shown to localise to the OLM in zebrafish^51^. The OLM is a semipermeable space that contains adherens junctions between the apical processes of Müller glia cells and the inner segments of photoreceptors^30^. The OLM is thought to act as a barrier that limits the diffusion of phototransduction cascade components, and structurally support photoreceptor motility in species with retinomotor movements^52^. Retinomotor movements involve the contraction or elongation of the microtubule-rich myoid in the photoreceptor inner segment, which influences the position of outer segments. This process is influenced by both the circadian clock and exposure to light^53^ and is mediated by cytoplasmic dynein in zebrafish, as genetic and pharmacological inhibition of dynein disrupts cone myoid elongation^51^. Based on its localisation in the OLM, circadian nature, and interaction with multiple dynein-related proteins during both the day and the night, clCRY2a/b is appropriately positioned to be involved in this process. Nevertheless it should be emphasised that the current evidence for retinomotor movement in birds is scant, but it may be to be a fruitful avenue of research in the future^54^.

## Limitations of the study

This study is limited by our ability to discriminate between clCRY2a and clCRY2b with our 2A3 antibody. Accordingly, we are not able to comment on the function of these different proteins or their subcellular localisation. To do so it would be necessary to generate two new antibodies, that distinguish between clCRY2a and clCRY2b. We expect that this would be challenging given they only differ in 12 amino acids.

## Methods

### Animal husbandry

Domestic rock pigeons (*Columba livia*) from our Austrian cohort were maintained on a 12L:12D light–dark cycle in a custom-built aviary, with the lights turning on at 07:00 Central European Time (CET). All experimental protocols were approved by the City of Vienna (Magistratsabteilung 58, GZ: 256824-2020-17) and the Austrian Federal Ministry of Science and Research (BMWFW-66.009/0211-WF/V/3b/2015). All methods were carried out in accordance with Austrian Animal Experiments Act. The study was performed in accordance with the ARRIVE guidelines.

### Cloning of pigeon cryptochrome 2a and 2b

To clone pigeon *clCRY2a* and *clCRY2b*, retinal cDNA libraries were created as previously described^21^. PCR primers were designed based on the annotated pigeon sequences for *CRY2a* and *CRY2b* using Primer 3 plus (http://www.bioinformatics.nl/cgi-bin/primer3plus/primer3plus.cgi). A high-fidelity, proofreading DNA polymerase (F-549S, Thermo Fisher Scientific) spiked with a non-proofreading polymerase (203205, Qiagen) was used to generate PCR products with 3′ A-overhangs suitable for TA cloning. The PCR products were cloned into a TOPO pCR 2.1 vector (K4520-01, Thermo Fisher Scientific) and analysed by Sanger sequencing.

### Laser microdissection, RNA isolation and real-time qPCR

Laser microdissection was performed as previously described^21^. Briefly, three pigeons were sacrificed by CO_2_ asphyxiation at approximately midday (12:00 CET). Under RNase-free conditions in a 4 °C room using DEPC-treated PBS, the right eye of each bird was removed, hemisected with a scalpel and the vitreous body was detached. The peripheral retina was cut away, and the central part of the retina (~ 5 mm in diameter) was isolated from the underlying choroid and sclera. The isolated retina was directly frozen in optimum cutting temperature (OCT) medium on dry ice and stored at − 80 °C until sectioning. 10 μm sections were mounted on RNAse-free polyethylene naphthalate membrane slides (11505189, Leica Microsystems), fixed in 70% ethanol, stained with Mayer’s haematoxylin (MHS32, Sigma-Aldrich) and dehydrated into 100% ethanol. For each slide, 20 pieces of a single retinal layer were dissected using the Leica LMD6500 system at 40× magnification (506208, Leica 40× dry PL-Fluota), and collected into a tube containing 35 μl of TRIzol lysis buffer (15596026, Thermo Fisher Scientific). The samples were snap-frozen in liquid nitrogen and stored at − 80 °C. For each biological replicate, three samples (each containing 20 pieces) from the corresponding retinal layer were pooled, and RNA was isolated by chloroform extraction and purified using the RNA Clean and Concentrator Kit (R1015, Zymo Research). Equal amounts of RNA from each bird was reverse transcribed to create cDNA with the Quantitect reverse transcription kit (205314, Qiagen). cDNA was diluted 1:5 in nuclease free water for qPCR analysis. For whole retina RNA isolation, three pigeons were sacrificed at midday and retinas were isolated as described above. Snap frozen retinas were homogenized with a TissueLyser II using 3-mm carbide beads (69997, Qiagen). Total RNA was extracted using the RNeasy Mini Kit (74104, Qiagen) and reverse transcribed using the QuantiTect reverse transcription kit (205314, Qiagen). cDNA was diluted 1:20 in nuclease free water for qPCR analysis. Exon spanning primers were designed using Primer3Plus and primer efficiencies were determined using a cDNA serial dilution series. Primer pairs with an efficiency of 95% to 105% were selected, and specificity was confirmed by analysis of melt curves (single peak) and Sanger sequencing of the amplified product (See Supplementary Table 5 for primer sequences). qPCR was performed in 96 well plates with 20 μl reactions using the 2× GoTaq Mastermix (A6001, Promega) on a CFX Connect Real-Time PCR Detection System (1855200, BioRad). Three internal control genes (*clHPRT*, *clGAPDH*, and *clTFRC*) and no template controls for each primer set were included on each plate. Gene expression was calculated relative to the geometric mean of the three control genes.

### Antibody production

The monoclonal antibody targeting clCRY2a/b (2A3) was generated in collaboration with the Ogris lab (Univ. Prof. Dr. Egon Ogris, Max Perutz Labs Vienna) as previously described^21^. The C-terminal region of C516-V583 is identical between CRY2a/b splice isoforms and was selected as the antigen. Codon-optimised mRNA for the CRY2a/b antigen was synthesised and cloned into the pET14b expression vector using the Genscript cloning service (GenScript USA Inc.) and then recombinantly expressed in *E. coli.* For purification, an N-terminal polyhistidine tag (6× His) was added to the antigen. As the CRY2 antigen was soluble in water, the lysate supernatants and were purified under non-denaturing conditions using a Nickel-sepharose column (17526801, GE Healthcare), and the Whatman Elutrap electroelution system (15560753, Thermo Fisher Scientific). BALB/c mice were immunised with the purified antigen and monoclonal antibody was produced using the hybridoma technique. The resulting fused hybridomas were selected by the HAT (hypoxanthine-aminopterin-thymidine) system, and the surviving cells were seeded in multi-well plates. The hybridoma cells from the 2A3 clone were weaned off selection media and then seeded into bioreactor flasks (Z688037-5EA, Sigma-Aldrich). The supernatant containing the secreted antibody was harvested and isolated by affinity purification using a Protein G column (28-9852-55, GE Healthcare) and then quantified by a NanoDrop 2000. Purified antibodies were stored at − 20 °C.

### Antibody validation by western blot

GFP-tagged recombinant clCRYs were expressed in primary cell cultures of pigeon embryonic fibroblasts (PEF) using the pCIneo expression vector (Promega, E1841). PEFs were cultured at 37 °C on sterile, uncoated 10-cm cell culture dishes (0030702115, Eppendorf). The culture medium was prepared with Dulbeccos Modified Eagles Medium (DMEM)—high glucose medium (MolBioService, IMP Vienna), supplemented with 1% penicillin-streptamycin (P0781, Sigma-Aldrich), 1% l-glutamine (G7513, Sigma-Aldrich), 1% non-essential amino acids (M7145, Sigma-Aldrich), 0.5% sodium pyruvate (S8636, Sigma-Aldrich), 6% fetal bovine serum (#10270-106, Gibco), and 8% chicken serum (C5405, Sigma-Aldrich). For transfection of a 10 cm dish, 20 μg of plasmid and 30 μl of Lipofectamine 2000 transfection reagent (11668, Invitrogen) were diluted in OptiMEM reduced serum medium (31985070, Gibco) according to the manufacturer’s instructions. The DNA-lipid complex was incubated for 10 min at room temperature and added to the cells. After 24 h, the transiently transfected cells were washed once with PBS and collected by trypsinization. 2 ml of a 0.15% Trypsin-PBS solution was added, and the cells were incubated at 37 °C for 5 min. The reaction was stopped by addition of 10 ml PEF medium, and the trypsinized cells were collected by centrifugation. The cell pellet was snap-frozen in liquid nitrogen, and lysed in RIPA buffer (150 mM NaCl, 1% Triton X-100, 0.5% sodium deoxycholate, 0.1% SDS, 50 mM Tris pH 8) supplemented with a protease inhibitor tablet before use (2187161, Pierce). For a single dish, 250–300 μl RIPA buffer was used. The cells were lysed by pipetting, incubated at 4 °C for 1 h and centrifuged for 30 min at 4 °C (16,200*g*). The supernatant was collected and stored at − 80 °C until use. For protein extraction from the pigeon retina, retinas from six individual birds were collected under normal light at midday (12:00 CET, n = 3) or under dim red light at midnight (00:00 CET, n = 3) and snap frozen in liquid nitrogen. Retina samples were resuspended in 400 µl of lysis buffer (20 mM tris (pH 7.5), 100 mM NaCl, 10% glycerol, 1% Triton X-100) with protease inhibitor and homogenized by a TissueLyser II (85300, Qiagen) for 2 min at 20 Hz. The lysates were centrifuged for 30 min at 4 °C (16,200*g*), and the supernatants were pooled. The amount of protein extract was determined using the BCA quantitation kit (23225, Pierce), and aliquots were stored at − 80 °C.

For SDS-PAGE, 20 μg of cell or retinal lysate was thawed on ice, and Bolt 4× LDS Sample Buffer (B0007, Thermo Fisher Scientific) and 10× reducing agent (B0004, Thermo Fisher Scientific) was added as per the manufacturer’s recommendation. The samples were heat denatured at 95 °C for 5 min and loaded into a Bolt 4 to 12% Bis–Tris gel (NW04127BOX, Thermo Fisher Scientific). The gel was run at 100–120 V in 1× MOPS buffer (B0001, Invitrogen) and samples were transferred onto a nitrocellulose membrane (GE Healthcare, 10600002) by wet electroblotting run at 80 V for 45 min and then 120 V for 45 min at 4 °C in transfer buffer (30 mM tris, 240 mM glycine, 0.025% SDS). A successful transfer was confirmed by Ponceau S staining. After de-staining in double deionised sterile H_2_O (ddH_2_O), membranes were blocked for 2 h at room temperature in 5% milk powder tris-buffered saline with 0.1% Tween (TBST). For the pre-adsorption control on cell lysates, clCRY2a/b 2A3 hybridoma supernatants were preincubated with 5 nmol (50–150 μg) of the 2A3 recombinant antigen for 2 h at room temperature under gentle agitation. After pre-absorption, the antibody-antigen solution was diluted to 1:50 in 5% milk/TBST. Membranes were cut into several pieces and exposed to either clCRY2a/b 2A3 hybridoma supernatant with or without antigen pre-absorption at 1:50 in 5% milk/TBST and were incubated overnight at 4 °C. The next day, the membranes were washed 3× TBST for 10 min and incubated for 1 h at room temperature with the HRP-tagged anti-mouse secondary antibody diluted 1:5000 in 0.5% milk-TBST (ab6823, abcam). Retinal lysate samples were treated as described above up to primary and secondary antibody treatment. Purified clCRY2a/b 2A3 was diluted 1:2000 in 2.5% milk/TBST and membranes were incubated overnight at 4 °C. After 6× TBST washes for 5 min, HRP-tagged anti-mouse secondary antibody was diluted 1:5000 in 2.5% milk/TBST and membranes were incubated for 1 h at room temperature. For detection, membranes were washed 3× TBST for 10 min and incubated with 1:1 of ECL (RPN2232, Cyriva) and ECL Select (RPN2235, Cytiva) for 90 s at room temperature. Excess fluid was removed, and the chemiluminescent signal was visualised with a ChemiDOC imager (17001402, BioRad).

### Immunohistochemistry

For immunohistochemical staining of recombinantly expressed clCRYs, neuroblastoma N2A cells were cultured at 37 °C on sterile, gelatine-coated glass coverslips in DMEM-high glucose medium supplemented with 10% fetal bovine serum (A3381901, Thermo Fisher Scientific), 1% l-glutamine (G7513, Sigma-Aldrich), and 1% penicillin–streptomycin (P0781, Sigma-Aldrich). Coverslips were sterilised by UV irradiation and coated with a 0.1% gelatine solution. N2A cells were grown overnight to 80% confluency. For transfection of clCRY-GFP constructs, 500 ng plasmid DNA and 2 μl Lipofectamine 2000 (11668027, Lifetech) prepared in Opti-MEM (31985062, Thermo Fisher Scientific) was added to the cells. After 5 h, the transfection medium was replaced with fresh N2A medium, and the cells were incubated overnight. To ensure sufficient expression of the GFP-tagged clCRYs, the eGFP tag was confirmed by fluorescent microscopy and the cells were fixed with 4% paraformaldehyde (PFA) for 15 min. After 3 washes in 1× PBS for 5 min the cells were permeabilised with 0.3% Triton-PBS for 5 min. Following 3 PBS washes for 5 min, the cells were blocked with 5% horse serum/1% bovine serum albumin in PBS (pH 7.3) for 15 min. The clCRY2a/b 2A3 hybridoma supernatant was diluted 1:500 in blocking solution and incubated with the cells for 1 h at room temperature. For antigen preincubation control experiments, tenfold excess by mass of the 2A3 antigen was added. After 3× PBS washes, the fluorescently conjugated secondary antibody (donkey anti-mouse Alexa 568; A10037, Thermo Fisher Scientific) was diluted 1:500 in blocking solution and added for 1 h at room temperature. The cells were counterstained with 4′,6-diamidino-2-phenylindole (DAPI) 1:1000 in 1× PBS (H3569, Thermo Fisher Scientific) and mounted in fluorescence mounting medium (S3023, Dako) before being imaged on a LSM800 upright confocal microscope.

For staining of retinal sections, pigeons were sacrificed at midday (n = 6) under normal light or at midnight (n = 3) under dim red light and the eyes were removed and hemisected. After extraction of the vitreous body, eyecups were fixed in 4% PFA for 20 min at room temperature and dehydrated in 30% sucrose in 1× PBS overnight at 4 °C. The retina was dissected and embedded in Neg-50 frozen section medium (6502, Thermo Fisher Scientific). 12 μm cryosections were mounted on SuperFrost Ultra Plus slides (J3800AMNZ, Thermo Scientific) and dried overnight at room temperature. After 1 PBS wash for 5 min, slides were incubated in antigen retrieval buffer (H-3301, Vector) gradually heated to 90 °C over 1 h. Slides were cooled down to room temperature and washed 3 times in PBS for 5 min. For permanent staining, slides were incubated in a humified chamber overnight at room temperature in 2A3 α clCRY2a/b (1:100) diluted in 0.3% Triton X-100/1× PBS with 4% dry milk powder. For antigen preincubation control experiments, tenfold excess by mass of the 2A3 antigen was added. After 3 PBS washes for 5 min, sections were incubated with ImmPRESS HRP-conjugated secondary antibody (MP-7402, Vector) for 1 h at room temperature and labelled with VIP purple (SK-4600, Vector). Images were captured on a Zeiss Axio Imager Z2 upright microscope. For fluorescent staining, slides were incubated in a humified chamber overnight at room temperature in primary antibody diluted in 0.3% Triton X-100/1× PBS with 2% donkey serum (ab7475, abcam). The following antibodies and concentrations were used: clCRY2a/b 2A3, 1:100 (mouse, 2.02 μg/μl); ZO-1, 1:100 (rabbit 61-7300, Thermo Fisher Scientific); calretinin, 1:500 (rabbit CR-7697, Swant). After 3× PBS washes for 10 min the next day, sections were incubated in the following fluorescently conjugated secondary antibodies 1:500 in 0.3% Triton X-100/1× PBS with 2% donkey serum for 2 h at 4 °C: donkey anti-mouse Alexa 647 (A-31571, Thermo Fisher Scientific); donkey anti-rabbit Alexa 568 (Thermo Fisher Scientific, A10042). Slides were washed 3 PBS for 10 min, counterstained with DAPI 1:1000 in 1× PBS (H3569, Thermo Fisher Scientific) and mounted in fluorescence mounting medium (S3023, Dako). Images were captured on a Zeiss LSM800 upright confocal microscope at various magnifications.

### Coimmunoprecipitation

Dynabeads coupled to protein G (10003D, Life Technologies) were crosslinked to purified α clCRY2a/b 2A3 antibody to precipitate clCRY2a/b and its potential interaction partners. As a negative control, beads crosslinked to α GFP 5G4 hybridoma supernatant were used. 100 μl of Dynabeads were equilibrated in 2 × 500 μl washes of Triton X-100/TBS (20 mM Tris pH 7.5, 150 mM NaCl + 0.04% Triton). 20 μg of α clCRY2 2A3 antibody was diluted in 500 μl Triton X-100/TBS. For the negative control, 250 μl of α GFP 5G4 hybridoma supernatant (5G4 raised against native GFP, Orgis lab) was diluted with 250 μl Triton X-100/TBS. Antibody solutions were added to equilibrated beads and incubated for 1 h rotating at room temperature. 3× Triton X-100/TBS washes were performed to remove unbound antibody. For covalent crosslinking, beads were washed 3× with 500 μl sodium borate solution (0.2 M sodium borate pH 9.2) and incubated with 1 ml of fresh DMP solution (20 mM dimethyl pimelimidate in 0.2 M sodium borate pH 9.2) for 30 min rotating at room temperature. To stop the cross-linking, beads were washed 2× for 10 min at room temperature with 500 μl Tris–HCl pH 8.0, then re-equilibrated with 2× washes in 500 μl Triton X-100/TBS. To remove non-crosslinked antibodies, 2× washes of 500 μl 0.1 M glycine (pH 2.0) were performed. Beads were re-equilibrated with 3× washes in 500 μl Triton X-100/TBS, transferred to Axygen Maximum Recovery tubes (MCT-175-L-C, Axygen) and stored at 4 °C overnight in 500 μl Triton X-100/TBS + 0.05% sodium azide. Pigeon retina samples were harvested and quantified at midday as described above (See *Antibody Validation by Western Blot*). Protein lysate was precleared by incubating with antibody free Dynabeads for 1 h at 4 °C. Precleared lysate was then incubated with α clCRY2a/b 2A3 crosslinked beads, or α GFP crosslinked beads for 30 min at 4 °C. The supernatant was removed and beads were washed 5 × 5 min with 800 μl ice cold wash buffer (20 mM Tris pH 7.5, 150 mM NaCl, 10% glycerol, 2 mM EDTA, 0.1% NP-40 + 1% Halt Protease and Phosphatase Inhibitor Cocktail EDTA-free (Thermo Scientific, 78445)) at 4 °C. To remove detergents, beads were washed 10× with 800 μl ice cold TBS (20 mM Tris pH 7.5, 150 mM NaCl) and 2× with 800 ml of 150 mM NaCl. During the last wash, the supernatant was removed, and pelleted beads were stored at − 80 °C.

### Mass spectrometry

For LC–MS/MS analysis, beads were resuspended in 50 μl of 100 mM ammonium bicarbonate (ABC), supplemented with 600 ng of lysyl endopeptidase (Lys-C, Fujifilm Wako Pure Chemical Corporation) and incubated for 4 h on a Thermo-shaker with 1200 rpm at 37 °C. The supernatant was transferred to a fresh tube and reduced with 1 mM Tris 2-carboxyethyl phosphine hydrochloride (TCEP, Sigma) for 30 min at 60 °C and alkylated in 5 mM methyl methanethiosulfonate (MMTS, Fluka) for 30 min at room temperature protected from light. Subsequently, the sample was digested with 600 ng trypsin (Trypsin Gold, Promega) at 37 °C over night. The digest was acidified by addition of trifluoroacetic acid (TFA, Pierce) to 1%. A similar aliquot of each sample (20%) was analysed by LC–MS/MS. The nano high performance liquid chromatography (HPLC) UltiMate 3000 RSLC nano system (Thermo Fisher Scientific) coupled to either a Q Exactive HF-X or Exploris 480 mass spectrometer equipped with a Nanospray Flex ion source (Thermo Fisher Scientific) was used to separate protein samples. Peptides were loaded onto a trap column (PepMap Acclaim C18, 5 mm × 300 μm ID, 5 μm particles, 100 Å pore size, Thermo Fisher Scientific) at a flow rate of 25 μl/min using 0.1% trifluoroacetic acid (TFA) as a mobile phase. After 10 min, the trap column was switched in line with the analytical column (PepMap Acclaim C18, 500 mm × 75 μm ID, 2 μm, 100 Å, Thermo Fisher Scientific). Peptides were eluted using a flow rate of 230 nl/min, and a binary linear 3-h gradient. The gradient started with the mobile phases 98% A (0.1% formic acid in water) and 2% B (80% acetonitrile, 0.1% formic acid), increased to 35% B over the next 180 min, followed by a steep gradient to 90% B for 5 min. After a 5-min hold, the gradient was ramped down over 2 min to the starting conditions of 98% A and 2% B for equilibration at 30 °C. For samples collected at midday (12:00 CET), the Q Exactive HF-X mass spectrometer was operated in data-dependent mode, using a full scan (m/z range 350–1500, nominal resolution of 60,000, target value 1E6) followed by tandem mass spectrometry (MS/MS) scans of the 10 most abundant ions. MS/MS spectra were acquired using normalized collision energy of 28, isolation width of 1.0 m/z, resolution of 30.000, target value of 1E5, maximum fill time 105 ms. Precursor ions selected for fragmentation (include charge states 2–6) were put on a dynamic exclusion list for 60 s. Additionally, the minimum AGC target was set to 5E3 and intensity threshold was calculated to be 4.8E4. The peptide match feature was set to preferred and the exclude isotopes feature was enabled. For samples collected at midnight (00:00 CET), the Orbitrap Exploris 480 mass spectrometer (Thermo Fisher Scientific) was operated in data-dependent mode, where two different FAIMS voltages were applied, performing a full scan (m/z range 350–1200, resolution 60,000 for − 45 FAIMS CV and 120,000 for − 60 FAIMS CV, normalized AGC target = 100%, MS1 = 1E6), followed each by MS/MS scans of the 10 most abundant ions. MS/MS spectra were acquired using a collision energy of 28, isolation width of 1.0 m/z, resolution of 45.000, normalized AGC target- 200%, MS2 = 2E5. Precursor ions selected for fragmentation (include charge state 2–6) were excluded for 45 s. For peptide identification of both midday and midnight datasets, the RAW-files were loaded into Proteome Discoverer (version 2.5.0.400, Thermo Fisher Scientific). All hereby created MS/MS spectra were searched using MSAmanda v2.0.0.16129^55^. RAW-files were searched against the National Center for Biotechnology Information (NCBI) pigeon genome using the following search parameters: The peptide mass tolerance was set to ± 10 ppm and the fragment mass tolerance to ± 8 ppm. The maximal number of missed cleavages was set to 2, using tryptic specificity with no proline restriction. Beta-methylthiolation on cysteine was set as a fixed modification, oxidation on methionine was set as a variable modification, the minimum peptide length was set to 7 amino acids. The result was filtered to 1% FDR on protein level using the Percolator algorithm^56^ integrated in Thermo Proteome Discoverer and was used to generate a smaller sub-database for further processing. The localization of the post-translational modification sites within the peptides was performed with the tool ptmRS, based on the tool phosphoRS^57^. Peptide areas were quantified using the in-house-developed tool apQuant^58^. Proteins were quantified by summing unique and razor peptides. Peptides that non-specifically bound to a control GFP antibody were excluded from further analyses.

### Data analysis and statistics

For STRING and GO analyses, putative clCRY2a/b interactors were ran through the STRING online tool (Version 11.0b, https://string-db.org/, STRING Consortium 2020^59^). Hits were mapped to corresponding proteins from *Homo sapiens* and were represented in the network with a high confidence interaction score (0.700) calculated with all active interaction sources. For ease of visualisation, disconnected nodes were hidden from the network. Colored edges denote evidence of protein–protein associations (See ‘*Legend’* in STRING). Functional enrichment analyses were performed in the STRING application program interface by mining the Gene Ontology (GO) database. GraphPad Prism (v8.0.2, GraphPad Software, San Diego, California USA, http://www.graphpad.com) was used to prepare graphs.

## Supplementary Information

Supplementary Information 1.

Supplementary Information 2.

Supplementary Information 3.

Supplementary Information 4.

Supplementary Information 5.
