# Supplementary material for: The expression, localisation and interactome of pigeon CRY2
Source: Sci Rep. 2021 Oct 13;11:20293. doi: 10.1038/s41598-021-99207-x (PMC8514597; doi:10.1038/s41598-021-99207-x)
Supplement: Supplementary file 5 — Supplementary Information 5. [file 41598_2021_99207_MOESM5_ESM.docx]

**Supplementary Figures**

**The expression, localisation and interactome of pigeon CRY2**

Spencer D. Balay, Tobias Hochstoeger, Alexandra Vilceanu, E. Pascal Malkemper, William Snider, Gerhard Dürnberger, Karl Mechtler, Stefan Schuechner, Egon Ogris, Gregory C. Nordmann, Lyubov Ushakova, Simon Nimpf, David A. Keays.

**
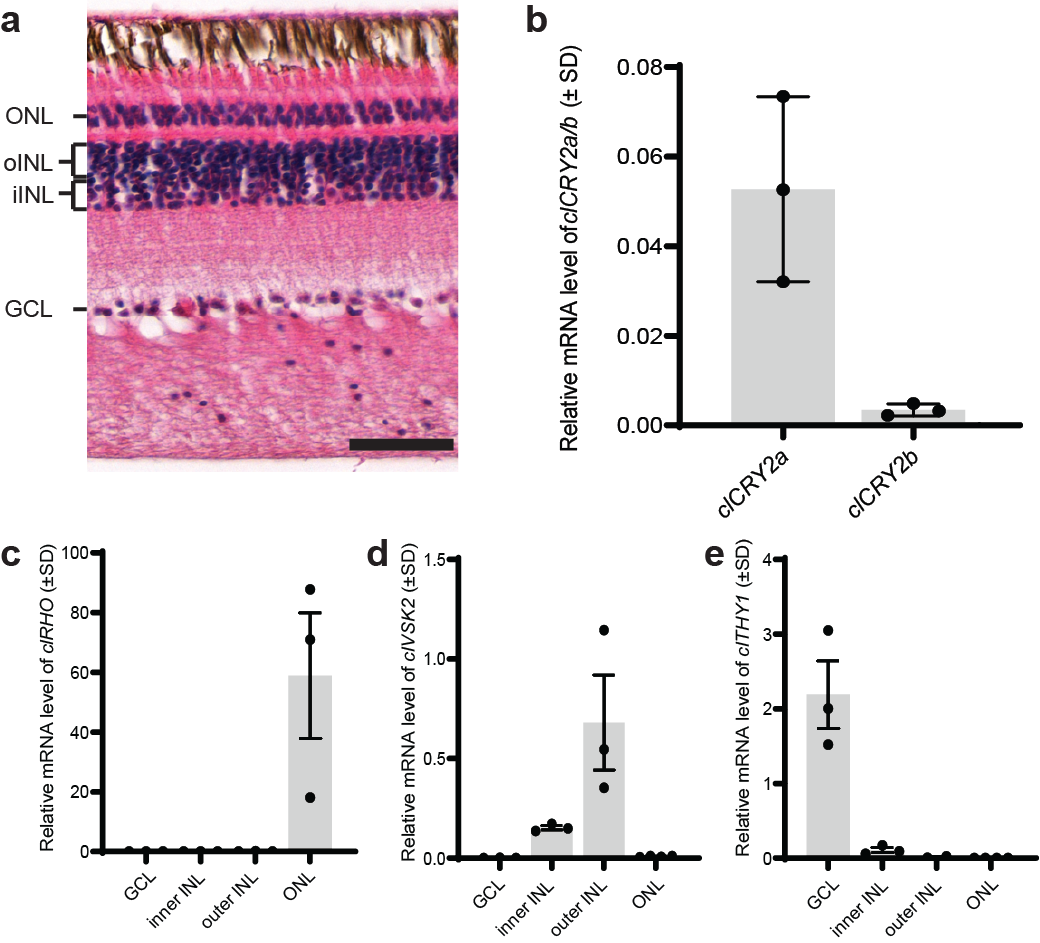
**

**Figure S1. Controls for *clCRY2a/b* laser dissection qPCR of pigeon retina. (a)** Pigeon retina stained with hematoxylin and eosin highlighting the outer nuclear layer (ONL), the inner nuclear layer (iINL), the outer inner nuclear layer (oINL), and the ganglion cell layer (GCL). **(b)** qPCR on whole pigeon retina reveals that *clCRY2a* is expressed approximately 15-fold higher than *clCRY2b* mRNA at midday **(c-e)** Expression levels of retinal layer control genes: ONL marker *clRHO* (C), INL marker *clVSX2* (D) and the GCL marker *clTHY1* (D). mRNA expression was normalized against the geometric mean of three control genes (*clHPRT*, *clGAPDH*, and *clTFRC*). Error bars: Standard Deviation (SD). Scale bar: 20 µm.


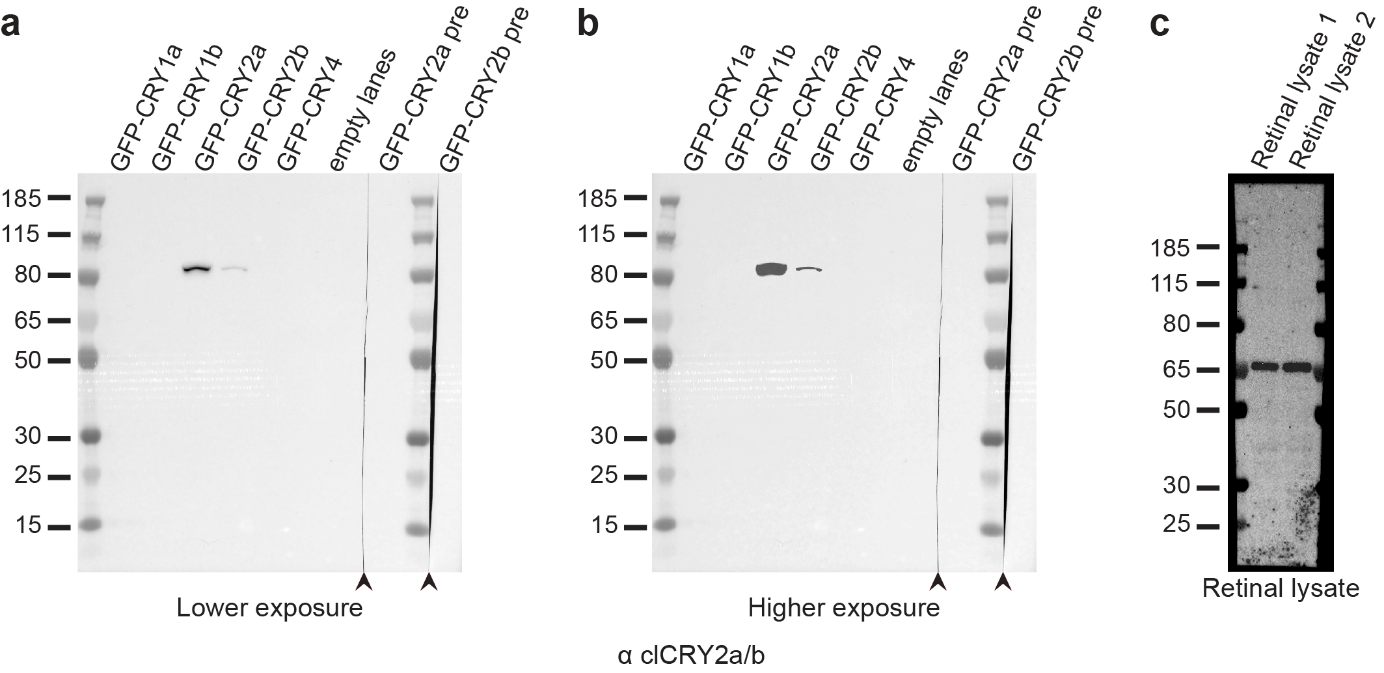


**Figure S2. Controls for clCRY2a/b western blot analysis. (a-c)** Uncropped images of blots used in Figure 2. **(a)** Image captured with lower exposure shows specific staining in pigeon embryonic fibroblast (PEF) cell lysates overexpressing GFP-tagged clCRY2a/b. Lanes used for pre-absorption controls were cut and incubated with the antigen separately before staining and detection. **(b)** The higher exposure blot was cropped for use in Figure 2A. Arrows highlight where blots were physically cut for antigen preincubation. **(c)** Uncropped retinal lysate blot shows specific recognition of a single approximately 67 kDa band in two independent retinal lysate samples. The first lane was cropped for use in Figure 2c.

**
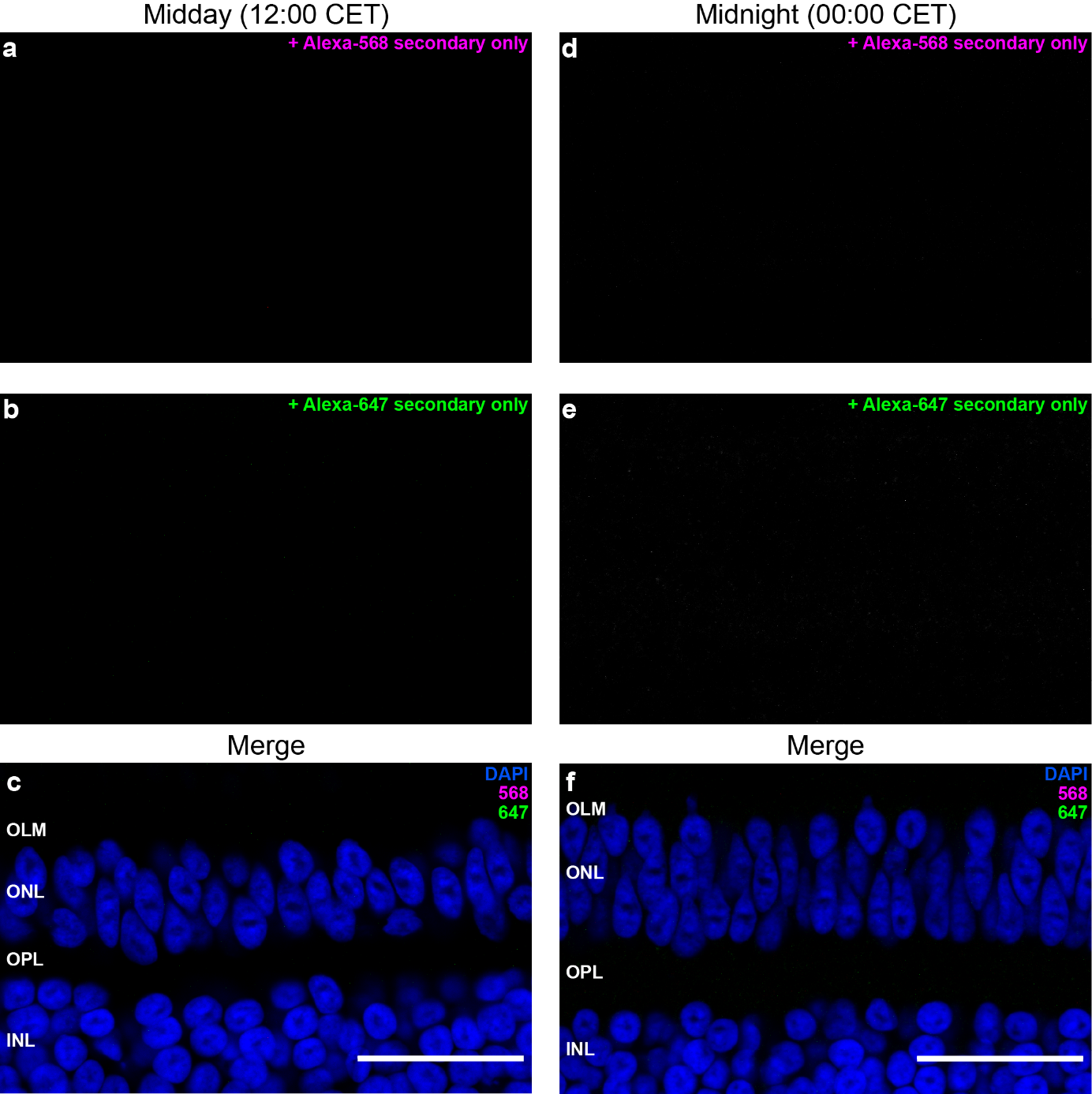
**

**Figure S3. Controls for clCRY2a/b immunofluorescence at midday and midnight. (a-f)** Pigeon retinal sections collected at midday (a-c) and midnight (d-f) treated with only secondary antibodies do not reveal detectable signal. Images were captured and processed the same as images in Figure 4a-f and Figure S3a-f. OLM: outer limiting membrane, ONL: outer nuclear layer, INL: inner nuclear layer, GCL: ganglion cell layer. Scale bars: 25 µm.

**
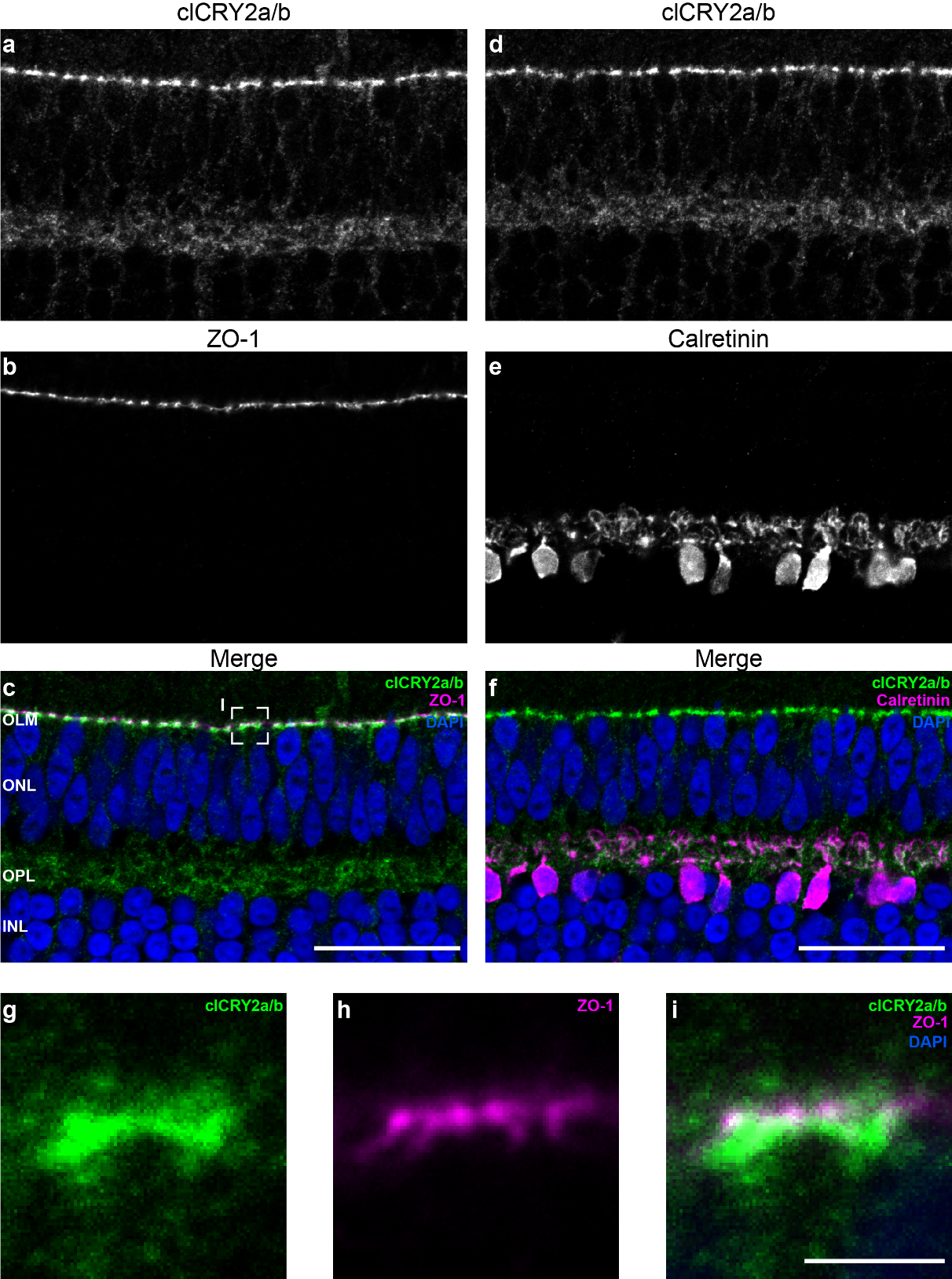
**

**Figure S4. clCRY2a/b is expressed in the OLM and OPL at midnight. (a-i**) Immunofluorescence staining reveals clCRY2a/b is expressed in the OLM (a-c) and OPL (d-f) at 00:00 CET. **(e-f)** Double staining with Calretinin shows that clCRY2a/b is expressed cytosolically in horizontal cells. **(g-i)** In the OLM, clCRY2a/b staining overlaps strongly with zonula occludens-1 (ZO-1), an adherens junction marker. OLM: outer limiting membrane, ONL: outer nuclear layer, OPL: outer plexiform layer, INL: inner nuclear layer. Scale bars: a-f: 25 μm, g-i: 3 μm. See Figure S3 for staining controls.

**
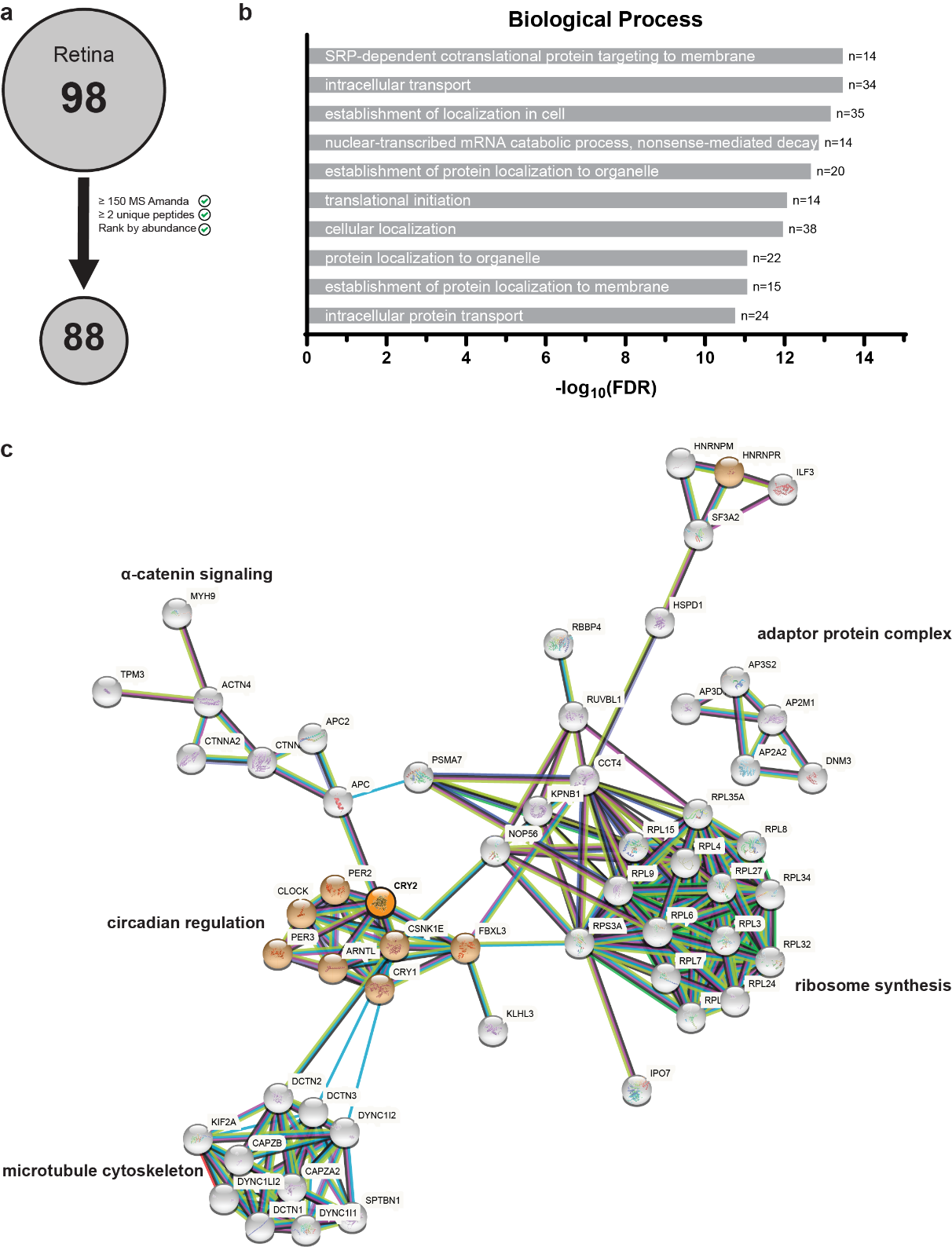
**

**Figure S5. Putative clCRY2a/b interaction partners at midnight. (a)** In total, 98 putative clCRY2a/b interactors that were not found in samples incubated with a control GFP antibody were pulled down with our 2A3 clCRY2a/b antibody in retinal lysates harvested at midnight (00:00 CET). Hits were ranked by average abundance and filtered for having an Amanda MS score of >150 and 2 or more unique peptides. After filtering, 88 hits were reliably identified in the retina. **(b)** Gene ontology (GO) analysis of clCRY2a/b and 87 putative clCRY2a/b interactors revealed an enrichment of proteins associated with multiple intracellular transport GO-terms. The top ten GO-terms based on FDR significance are shown. **(c)** STRING analysis (Version 11.0b, <https://string-db.org/>, STRING Consortium 2020) shows clCRY2a/b interactors cluster in four distinct groups similar to the midday dataset in Figure 5. Hits containing the circadian GO-terms highlighted in Figure 5b are colored orange.

| **Supplementary Table 5. Primers for qPCR analysis** | | |  |
| --- | --- | --- | --- |
| Transcript | Forward primer | Reverse primer |  |
| *clCRY2a* | GGAAGAGCTGGGCTTTCCTAC | TTCTTTCCAAGTGTTTATCCAGCC |  |
| *clCRY2b* | TGTAAAGTGGACATCCAGGAGAAC | ATTTGAAGCCATGCCCAGCTC |  |
| *clTHY1* | CTACACTGGCAACCAGATAAAGAA | GTTCTGGATCAAGAGGCTGAAG |  |
| *clVSX2* | ACAATCTTCACATCCTACCAACTG | GAACCAAACCTGTATCCTGTCTTC |  |
| *clRHO* | GTTCTACATCTTCACCAACCAGG | CAGAGAGTTGTGATCATGCAGTTA |  |
| *clHPRT* | CTCCTCGAAGTGTGG | AGTATTCATTGTAATCTAGGG |  |
| *clGAPDH* | TCAATGGGAAACTTACTGGAATGG | TCTTAATGTCATCATACTTGGCTGG |  |
| *clTFRC* | CATATTATTGGAACTTGCCCGTGT | CCACAGCTCCATACTCTCCT |  |
